# New Functions of Vav Family Proteins in Cardiovascular Biology, Skeletal Muscle, and the Nervous System

**DOI:** 10.3390/biology10090857

**Published:** 2021-09-01

**Authors:** Sonia Rodríguez-Fdez, L. Francisco Lorenzo-Martín, Salvatore Fabbiano, Mauricio Menacho-Márquez, Vincent Sauzeau, Mercedes Dosil, Xosé R. Bustelo

**Affiliations:** 1 Molecular Mechanisms of Cancer Program, Centro de Investigación del Cáncer, CSIC-University of Salamanca, 37007 Salamanca, Spain; soniarf@usal.es (S.R.-F.); Fran_lm@usal.es (L.F.L.-M.); s.fabbiano@cell.com (S.F.); mmenacho@conicet.gov.ar (M.M.-M.); vincent.sauzeau@inserm.fr (V.S.); mdosil@usal.es (M.D.); 2 Instituto de Biología Molecular y Celular del Cáncer, CSIC-University of Salamanca, 37007 Salamanca, Spain; 3 Centro de Investigación Biomédica en Red de Cáncer (CIBERONC), CSIC-University of Salamanca, 37007 Salamanca, Spain; 4 Instituto de Inmunología Clínica y Experimental, CONICET, Rosario 3100, Argentina; 5 Institut du Thorax, UMR1087 CNRS 6291, INSERM, Université de Nantes, 44096 Nantes, France

**Keywords:** vascular smooth muscle cells, skeletal muscle cells, neurons, glia, ventrolateral medulla, nitric oxide, protein tyrosine kinases, cytoskeleton, signaling, receptor internalization, sympathetic nervous system, blood pressure, vasoconstriction, vasodilation, hypertension, axon wiring, myelination, metabolic syndrome, type II diabetes, obesity

## Abstract

**Simple Summary:**

In this review, we provide information on the role of Vav proteins, a group of signaling molecules that act as both Rho GTPase activators and adaptor molecules, in the cardiovascular system, skeletal muscle, and the nervous system. We also describe how these functions impact in other physiological and pathological processes such as sympathoregulation, blood pressure regulation, systemic metabolism, and metabolic syndrome.

**Abstract:**

Vav proteins act as tyrosine phosphorylation-regulated guanosine nucleotide exchange factors for Rho GTPases and as molecular scaffolds. In mammals, this family of signaling proteins is composed of three members (Vav1, Vav2, Vav3) that work downstream of protein tyrosine kinases in a wide variety of cellular processes. Recent work with genetically modified mouse models has revealed that these proteins play key signaling roles in vascular smooth and skeletal muscle cells, specific neuronal subtypes, and glia cells. These functions, in turn, ensure the proper regulation of blood pressure levels, skeletal muscle mass, axonal wiring, and fiber myelination events as well as systemic metabolic balance. The study of these mice has also led to the discovery of new physiological interconnection among tissues that contribute to the ontogeny and progression of different pathologies such as, for example, hypertension, cardiovascular disease, and metabolic syndrome. Here, we provide an integrated view of all these new Vav family-dependent signaling and physiological functions.

## 1. Introduction

The Vav family is a group of signal transduction molecules that work as GDP/GTP exchange factors (GEFs) for GTPases of the Rho subfamily and, in some other cases, as adaptor-like molecules. This family is composed of single representatives in invertebrates (generically designated as Vav proteins in each species) and three members in most vertebrates (Vav1, Vav2, Vav3). However, depending on the phylogenetical stage, reduced and increased numbers of Vav family proteins can be found in some species [1,2]. The first member of this family was discovered in Mariano Barbacid’s lab in 1989 due to the spurious stimulation of its transforming activity during transfections of a human tumor genomic DNA in NIH3T3 cells [3]. Given that this oncogene was the sixth one isolated in that lab, it was named as the sixth letter of the Hebrew alphabet (*Vav*). The Vav2 and Vav3 proteins were identified from 1995 to 2000 [4,5,6]. The explosion of genomic data derived from genome sequencing efforts subsequently led to the discovery of the rest of the Vav family members present in both vertebrate and invertebrate species [1].

**Figure 1:**
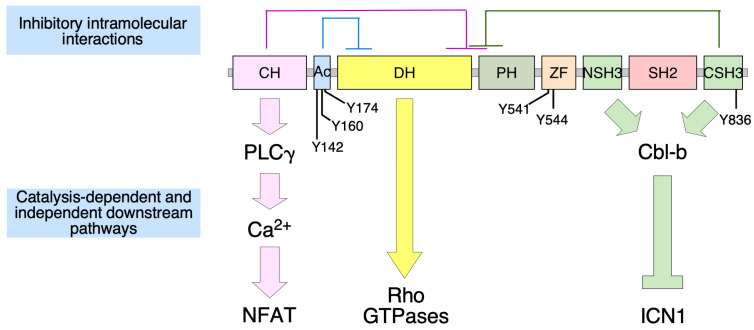
Depiction of the structure, the main regulatory phosphosites (using the amino acid sequence corresponding to mouse Vav1), the intramolecular autoinhibitory interactions that take place in the nonphosphorylated state of Vav proteins (top), and some of the main downstream pathways described for specific mammalian Vav family members (bottom). NFAT, nuclear factor of activated T cells (a transcriptional factor involved in the proliferation and cytokine production of T lymphocytes); ICN1, intracellular domain of Notch1. The rest of abbreviations are described in the main text.

Mammalian Vav proteins harbor eight structural domains associated with regulatory and/or effector proteins (Figure 1). These domains include calponin-homology (CH), acidic (Ac), Dbl-homology (DH), pleckstrin-homology (PH), C1 subtype zinc finger (ZF), noncanonical SH3 (NSH3), SH2, and canonical SH3 (CSH3) regions. The catalytic activity of these proteins is mediated by the catalytic DH domain in a concerted action with the PH and ZF regions, a rather unique feature of this protein family compared with the rest of Rho GEFs [1,2,5,7,8,9]. However, Vav proteins can also activate additional downstream pathways using catalysis-independent mechanisms. These adaptor functions are in many cases Vav family member- and cell type-specific (Figure 1). For example, Vav1, but not Vav2, can activate the nuclear factor of activated T cells using a CH-dependent mechanism in T lymphocytes [10,11,12]. Likewise, Vav1 plays tumor suppressor roles in T cell acute lymphoblastic leukemia using an adaptor mechanism mediated by its SH3 domains. This function promotes the ubiquitin-mediated degradation of the active, intracellular fragment of Notch 1 by forming complexes with the E3 ubiquitin ligase Cbl-b (Casitas B-lineage lymphoma b) [13]. The SH3 regions of Vav proteins can bind to many other protein partners, although their specific downstream role is not understood as yet. However, these interactions suggest that Vav proteins might participate in additional scaffold-like functions in cells.

Vav proteins require phosphorylation by upstream protein tyrosine kinases to become activated [2]. This is due to inhibitory interactions established by the N-terminal (CH and Ac region) and the C-terminal (CSH3 domain) regions with the catalytic core when the proteins are in the nonphosphorylated state (Figure 1). These interactions, which occlude the effector sites of these proteins, are eliminated upon the phosphorylation of Vav proteins on specific tyrosine residues (Figure 1). This leads to the stimulation of the catalytic activity and most adaptor functions upon the exposure of the effector sites [2]. The structural basis for the autoinhibition of Vav proteins by the CH-Ac has been clarified due to the resolution of the crystal structure of a large N-terminal fragment of Vav1 [14]. The CSH3-mediated intramolecular inhibition has been inferred using biochemical, signaling, and cell biology experiments. However, it is worth noting that a model for the inhibitory action of the CSH3 has recently been identified for Vav2 and Vav3 using artificial intelligence approaches (see the AlphaFold Protein Structure Database, entries at https://alphafold.ebi.ac.uk/entry/P52735 (accessed on 30 July 2021) and https://alphafold.ebi.ac.uk/entry/F1LWB1, accessed on 30 July 2021). The regulation of Vav proteins by tyrosine phosphorylation is rather unique in the Rho GEF family. Underscoring this issue, Vav proteins are the only Rho GEFs that contain the phospho-tyrosine binding SH2 region. Recent data have revealed that the signaling output of Vav proteins can be regulated by additional mechanisms such as acetylation, binding to plasma membrane-resident phosphatidylinositol monophosphates, and expression [1,2,15,16].

Accumulating evidence indicates that Vav proteins play critical roles in a wide range of physiological and pathological processes. Some of these signaling functions have been previously reviewed [1,2]. Detailed information on the phylogenetic origin of the Vav family is also available from a previous publication [1]. Additional information on Vav functions will be found in review articles that, together with the present one, form part of the Special Issue on Vav proteins published by this journal. Here, we will specifically aim at providing an update of what is known about the role of these proteins in cardiovascular biology, skeletal muscle, and different branches of the nervous system. As a note of caution, we will exclusively focus on signaling and physiological mechanisms that have been corroborated using mouse models. As we will see, the study of Vav proteins in these functions has allowed us to better understand both the regulation and roles of these proteins. In addition, it has illuminated new layers of the ontogeny and progression of complex diseases such as metabolic syndrome and hypertension.

## 2. Vav2, Vascular Smooth Muscle Cells, and Cardiovascular Regulation

**Figure 2:**
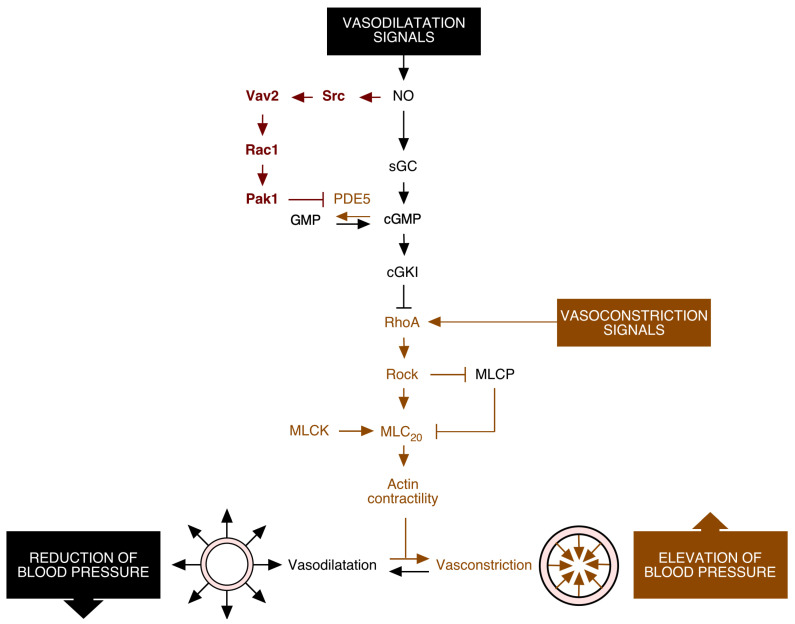
Schematic representation of the Vav2–Rac1-dependent pathway promoting blood vessel vasodilatation (red color). The previously known signaling elements of the NO signaling route in vSMCs are shown in black. The vasoconstriction pathway is shown in brown. Activation steps are indicated by arrows. Inhibitory steps are indicated by blunted lines. See further details in main text. Abbreviations used are: sGC, soluble guanylate cyclase; cGKI, cGMP–dependent protein kinase type I; GMP, guanosine monophosphate; MLCK, MLC_20_ kinase. The rest of the abbreviations have already been described in the main text.

The control of systemic blood pressure is ensured, among other physiological responses, by regulating the contractility of blood vessels in real time. A key signal for this process is nitric oxide (NO) [17,18], a vascular endothelial cell-generated gas that favors the reduction in blood pressure via the induction of the vasodilatation or resistance arterioles. This process entails the disassembly of the F-actin cytoskeleton, of protein nitrosylation events, and of other signaling regulatory steps in NO-stimulated vascular smooth muscle cells (vSMCs) [17]. To induce the former response, NO promotes the step-wise stimulation of soluble guanylate cyclase [17,19], production of cyclic guanosine monophosphate (cGMP) [17,19], and the enzyme activity of the cGMP-dependent protein kinase type I [17,19,20] (Figure 2, pathway in black color). The latter enzyme triggers the phosphorylation of the RhoA GTPase that, in turn, causes the release of the GTPase from the plasma membrane and its disassembly from the downstream serine/threonine kinase Rock1 (Rho-associated coiled-coil-containing protein kinase 1) [21]. This leads to the vasodilatation-mediated reduction in blood pressure because the inactivation of Rock1 abrogates stress fiber formation via the concerted down- and upregulation of the 20 kDa myosin light chain (MLC_20_) and the MLC_20_ phosphatase (MLCP), respectively [22,23,24] (Figure 2). This NO-regulated vasodilatation pathway is negatively regulated by phosphodiesterase type 5 (PDE5), an enzyme that hydrolyzes cGMP [25] (Figure 2). The importance of this route in normal physiology is demonstrated by the observation that the inactivation of several signaling elements of this pathway using either genetic or pharmacological avenues leads to the rapid development of a hypertensive state [20,26,27,28,29,30]. Conversely, the use of PDE5 inhibitors (e.g., sildenafil, the active component of Viagra) restores most vasodilatation defects associated with hypertensive states and erectile dysfunction [25,31,32]. While this pathway had been known for a long time, the investigation of the cause of the hypertension exhibited by Vav2-deficient mice [33] allowed for the discovery of a new signaling branch that cooperates with the previously known pathway to favor the dilatation of resistance arterioles (Figure 2, pathway in red) [34]. This branch requires the Src-dependent phosphorylation and activation of Vav2 upon the stimulation of vSMCs with NO (Figure 2). Activated Vav2 leads, in turn, to the stimulation of Rac1, the Rac1-mediated translocation of the serine/threonine kinase Pak (p21–activated kinase), and the Pak1-mediated inactivation of PDE5 (Figure 2) [34]. This inhibitory step unexpectedly relies on the physical interaction of Pak1 with the N-terminal domains of PDE5, rather than on a transphosphorylation-dependent step. It is hypothesized that this inhibition step involves a conformational change in the target protein [34], given that the PDE5 N-terminus contains domains involved in both the homodimerization and the upstream regulation of PDE5 enzymatic activity [35]. The inhibition of PDE5 by Vav2 therefore ensures high levels of cGMP production in NO-stimulated vSMCs, thus favoring the sustained silencing of the RhoA pathway, effective vasodilation, and reduction in blood pressure (Figure 2) [34]. Further genetic evidence has demonstrated that this pathway is intrinsic to vSMCs and is dependent on the main Vav2 substrate, the GTPase Rac1 [36,37]. These results indicate that Vav2 is a natural “Viagra”-like molecule that ensures proper NO-triggered vasodilatation responses by limiting the enzyme activity of PDE5. Consistent with this idea, the development of the hypertension and its associated cardiovascular comorbidities can be prevented in *Vav2*^–/–^ and vSMC-specific *Rac1*^–/–^ mice using sildenafil treatments [34,36].

## 3. Vav2, Skeletal Muscle, and Metabolic Homeostasis

The analysis of the role of Vav2 in skeletal muscle and associated physiological mechanisms has been made using second-generation *Vav2*^L332A^ and *Vav2*^Onc^ knock-in mouse models. The former strain expresses a version of Vav2 with a point mutation in the DH region (Leu^332^ to Ala) that reduces its catalytic activity by approximately 70% [38]. The latter strain expresses an N-terminally truncated (residues 1 to 186) version of the protein that showed catalytic hyperactivity due to the elimination of the inhibitory CH and Ac regions (Figure 1) [36]. Thus, these two mouse models allowed us to address, for the first time, the contribution of the deregulated catalytic activity of Vav2 to a specific biological process. Using these models, we found that Vav2 signaling is critical for the control of skeletal muscle mass due to its implication in the regulation of the optimal output from the phosphatidylinositol 3 kinase α (PI3Kα)–Akt axis upon the stimulation of skeletal muscle cells with either insulin or IGF1 (insulin growth factor 1) [39]. Consistent with this, it was observed that homozygous *Vav2*^L332A/L332A^ and *Vav2*^Onc/Onc^ mice showed reduced and increased muscle mass, respectively [39]. This is an intrinsic function of Vav2 in skeletal muscle cells, since the signaling alterations can be recapitulated using both loss- and gain-of-function approaches in cultured skeletal muscle cells [39]. The signaling dissection of this pathway indicated that Vav2 contributes to the activation of the PI3Kα–Akt axis using GTPase Rac1 as the main substrate [39].

The skeletal muscle is also responsible for ≈80% of the glucose uptake and clearance induced by insulin at the whole body level [40,41]. It also regulates the physiological status of other tissues involved in metabolic homeostasis such as the brown (BAT) and white (WAT) adipose tissue through hormonal-mediated mechanisms [40,42,43]. As a result, signaling dysfunctions in skeletal muscle cells can cause the development of type 2 diabetes and metabolic syndrome in both mice and humans [40,41]. It is not surprising, therefore, that *Vav2*^L332A/L332A^ mice also showed a progressive increase in adiposity in both the BAT and WAT, which eventually caused the subsequent development of liver steatosis and hyperglycemia [39]. These problems are accelerated, and further aggravated, when these mice are maintained under high-fat diet conditions [39]. Conversely, *Vav2*^Onc/Onc^ animals exhibited resistance against the foregoing dysfunctions when subjected to a high-fat diet [39]. These metabolic alterations are quite similar to those previously found in other genetically modified mouse models that display either reduced (as in *Vav*2^L332A^ mice) or increased (as in *Vav2*^Onc^ mice) skeletal muscle mass [44,45,46,47,48,49,50].

## 4. Vav2 and Neuronal Functions

Vav2 plays roles in signaling processes related to the internalization of transmembrane receptors in the nervous system. The first example of these functions was given by Greenberg’s group in 2005, when they discovered that Vav2 is important for the regulation of the internalization of Eph family receptors in specific neuronal subtypes [51]. This function is important for proper growth collapse and the correct establishment of axon projections from retinal ganglion neurons to cells located in the dorsal geniculate nucleus [51].

Vav2 is also involved in the endocytosis of Ret–dopamine transporter complexes present in neurons of the nucleus accumbens [52], a part of the mesolimbic pathway of the brain that becomes stimulated during rewarding experiences and the intake of some drugs [53]. In fact, the dopamine transporter is the molecular target for cocaine [53]. Loss of Vav2 leads to the accumulation of the dopamine transporter in plasma membrane and an increase in the intracellular levels of dopamine in mice [52]. This function is nucleus accumbens-specific, since the lack of Vav2 does not affect the overall dopamine content of other midbrain regions involved in the mesolimbic pathway. It is also Vav2-specific, as *Vav3*^–/–^ mice do not display any defects in dopamine levels in any of those midbrain regions [52]. Interestingly, the effects of cocaine are severely reduced in the absence of Vav2 [52]. This has been connected to reductions in the dopamine transporter *K*_m_ and to smaller amplitude of the elevation of dopamine levels in the nucleus accumbens in the presence of cocaine. In contrast, no overt changes in the mesolimbic pathway-associated behaviors have been found under normal conditions in *Vav2*^–/–^ mice [52].

## 5. Neuron-Associated Vav3 Functions in the Brainstem, Cerebellum, and Retina

**Figure 3:**
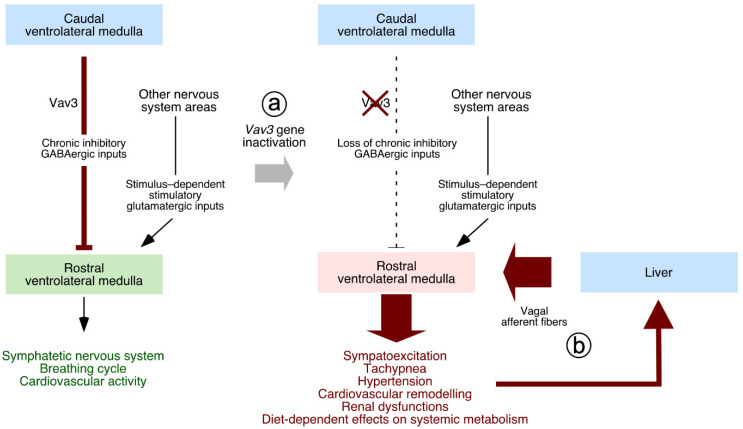
Involvement of Vav3 in the regulation of sympathetic output from the RVLM. *Left*, normal conditions. *Right*, sympathoexcitation-induced physiological alterations found in Vav3-deficient mice (**a**). The influence of peripheral stimuli, transmitted to the RVLM using the vagus nerve is shown in (**b**) (see Section 7). Further details are discussed in the main text.

Unlike the case of Vav2, the elimination of the *Vav3* gene causes widespread physiological alterations in mice due to severe problems in the regulation of the sympathetic nervous system (SNS). This is due to the implication of Vav3 in the establishment of proper inhibitory GABAergic wiring between the caudal (CVLM) and the rostral (RVLM) ventrolateral medullas that are located in the brainstem area [54] (Figure 3, point a). This wiring is essential for proper sympatho-regulation, since the CVLM is in charge of feeding tonic inhibitory signals to the RVLM and, at the same time, relay afferent signals from peripheral baroreceptors [55,56,57,58,59]. These activities are required, for example, to ensure the rapid restoration of normotensia upon sporadic changes in blood pressure. The CVLM also contributes to reset the threshold for the activation of the baroreflex by RVLM cells, an action that facilitates adaptative rises of blood pressure to new environmental, health, or physiological conditions [55]. The migration of axons of GABAergic neurons located in the CVLM toward their target neurons of the RVLM is impaired in the absence of Vav3 (Figure 3, point a) [54], leading to the unleashing of RVLM activity, the hyperactivation of the SNS, and the development of SNS-dependent defects such as hypertension, tachypnea, and hypercapnia (Figure 3, point a) [54,60]. All these dysfunctions can be prevented or reverted when treating the animals with β-adrenergic antagonists such as propranolol [54,60]. Interestingly, similar SNS-dependent dysfunctions are detected in mice lacking Ahr [61], a transcriptional factor that regulates Vav3 expression [62].

**Figure 4:**
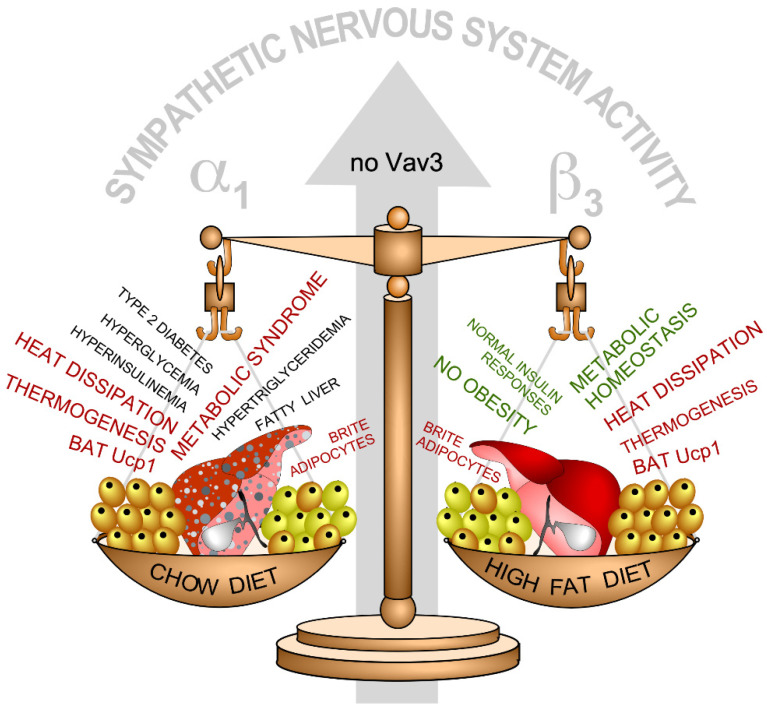
Summary of the physiological and metabolic dysfunctions found in *Vav3*^–/–^ mice under chow (left) and high-fat (right) diet conditions in BAT (cells colored in light brown), WAT (cells colored in yellow), liver (red), and plasma. Defects are shown in red and black. Normal responses are shown in green. The main adrenergic receptors involved in the responses found in chow (α_1_) and high-fat diet (β_3_) conditions are indicated. Further information is in the main text and reference [63].

The SNS hyperactivity is also the original cause of post-receptor insulin-like state and the obesity-independent metabolic syndrome seen in chow diet-fed *Vav3*^–/–^ mice (Figure 4) [63]. Interestingly, this metabolic phenotype is highly dependent on the type of diet because, unexpectedly, the Vav3-deficient animals do not develop the foregoing alterations when maintained under a high-fat diet regimen (Figure 4) [63]. Even more unexpectedly, these mice are totally protected against obesity and the ensuing metabolic syndrome condition that typically develops in mice under high-fat diets (Figure 4) [63]. Several physiological processes contribute to this paradoxical metabolic phenotype. On one hand, the protection from obesity exhibited by these animals independently of the diet used is the result of the presence of constitutive thermogenic programs in the BAT and the WAT that are sustained through adrenergic signals conveyed by both β_3_ and α_1_ receptors (Figure 4) [63]. On the other hand, the metabolic syndrome that develops in *Vav3*^–/–^ mice is caused by two separate, SNS-dependent inputs on the liver: (a) An extrinsic effect elicited by peripheral tissues that promotes a post-receptor insulin state, de novo lipogenesis, and liver steatosis in *Vav3*^–/–^ mice, regardless of the type of diet used [63]; and (b) an intrinsic effect on the liver itself that causes the upregulation of Pgc1α, a transcriptional cofactor involved in the activation of gluconeogenic, fatty acid oxidation, and ketogenic routes during fasting responses [63]. All these data indicate that the loss of Vav3 in the CVLM causes a butterfly-like effect that leads to the progressive alteration of many SNS-dependent physiological processes at the whole organismal level.

Outside the VLM, the *Vav3* gene deficiency causes a retardation in the developmental steps of cerebellar Purkinje and granule cells. These defects lead to transient motor coordination and gaiting defects in very young *Vav3*^–/–^ mice [64]. At the cell biology level, it has been demonstrated that Vav3 regulates the branching of dendrites in both Purkinje and granule cells in culture [64]. The cerebellar phenotype of Vav3-deficient animals is similar to those found in animals lacking BDNF (brain-derived neurotrophic factor), NT3 (neurotrophin 3), and calcyphosine 2 [65,66,67]. BDNF and NT3 are ligands for TrkB (tropomyosin receptor kinase B) [68]. Calcyphosine 2 is an intracellular calcium-binding protein that participates in the secretion of the foregoing neurotrophins and, thereby, for the local bioavailability of these ligands to the TrkB-expressing cells located in the cerebellum [67]. This phenotypic similarity suggests that some of the defects found in Vav3-deficient mice could be related to the defective stimulation of the neurotrophin–TrkB signaling axis. In agreement with this idea, *Vav3*^–/–^ knockout mice displayed low levels of BDNF than the controls within specific cerebellar areas during perinatal ages [64]. Given the transient nature of the cerebellar phenotype observed in *Vav3*^–/–^ mice, it is likely that other Rho GEF could carry out functions analogous to Vav3 during this process concurrently or at later postnatal ages. Obvious candidates include GEFs, whose elimination causes cerebellar defects such as P-Rex family members, Trio, β-Pix, and Dock10 [69,70,71,72,73].

Finally, the genetic elimination of Vav3 enhances the differentiation of early neuronal lineages such as ganglion and cone photoreceptor cells in the retina of mouse embryos. This alteration, however, is corrected later on in postnatal stages [74]. It is likely that this process is mediated by the regulation of Vav3 expression and, subsequently, the stimulation by extracellular ligands for transmembrane tyrosine kinase receptors. Potential candidates for this stimulation step include ligands for both the epidermal and fibroblast growth factor receptors [74].

## 6. Vav3, Oligodendrocytes, and Myelination Processes

Similar to the case of retinal cells [74], the elimination of Vav3 accelerates the differentiation of oligodendrocytes in mice [75]. The *Vav3* gene deficiency also delays, although it does not abrogate the myelination of fibers in both cortical and cerebellar areas [75]. This process seems to be Rho GTPase-dependent, although the upstream and downstream mechanism involved still remain to be elucidated [75]. The physiological consequences of these defects are also unknown.

## 7. Lessons Learnt from the Phenotypes of Vav Family Knock-Out and Knock-In Mice

Together with the identification of new Vav family-regulated signaling and physiological processes, the study of the mouse models for the Vav family has unveiled new data on the regulation of the Vav proteins themselves as well as on the biological programs they participate in. In the former case, for example, we have learnt that the phosphorylation-mediated activation of Vav proteins can be mediated not only by antigens and ligands for specific tyrosine kinase receptors, but also by NO (Figure 2) [34]. Despite this, the activation of Vav2 by this gas still requires the expected participation of protein tyrosine kinases, in this case, of the cytoplasmic Src family [34]. The exact mechanism that connects NO with those kinases is as yet unknown. In the latter case, the analysis of the Vav family-regulated physiological programs has also shed light on lingering questions affecting a number of etiological factors and cross-talk that are associated with the development of cardiovascular and metabolic diseases. For example, a rather obscure issue in this field is the role played by chronic sympathoexcitation in the development of both obesity and metabolic disease. Likewise, the role of hypertension in the development of type II diabetes and the ensuing metabolic syndrome is under debate. Answering these questions is highly relevant, since the data obtained will give us information on whether all these pathologies are etiologically and mechanistically intertwined or whether they just develop concurrently (but independently from a mechanistic point of view) as a consequence of the lifestyle of people. Tackling these issues from a clinical point of view has been rather difficult up to now given the multiple ethnic, sex, genetic, and environmental layers that affect the origin and evolution of all these illnesses. The availability of *Vav3*^–/–^ mice has made it possible to use them as genetically “clean” tools to dissect the contribution of chronic sympathoexcitation to these pathologies. The findings obtained with them indicate that, at least in the case of rodents, the chronic stimulation of the SNS does affect the development of most pathological dysfunctions linked to metabolic syndrome conditions (Figure 4) [63]. They also suggest that treatments with α-adrenergic receptor antagonists, but not with β-adrenergic receptor antagonists, can be utilized to eliminate the metabolic syndrome condition in non-obese individuals displaying chronic sympathoexcitation (Figure 4) [63]. The combined used of *Vav2*^–/–^ and *Vav3*^–/–^ mice also demonstrated that hypertension does not contribute *per se* to the development of type II diabetes and metabolic syndrome [63]. 

The use of *Vav3^–/–^* mice led to the discovery that vagal signals originated from peripheral tissues are essential for the consolidation of the systemic pathologies caused by RVLM-driven sympathetic hyperactivity [76]. In line with this, it was observed that the surgical or chemical elimination of the afferent vagal branch that goes from the liver to the brainstem eliminates all the cardiovascular and metabolic defects found in Vav3-deficient animals [76] (Figure 3, point b). The transmission to, and subsequent integration of these vagal nerve-transmitted inputs into the RVLM requires further investigation.

Finally, there is the issue of whether the functions discovered in mice have a translation to human settings. At moment, this question remains to be tackled. However, it is worth noting that genetic association studies have found associations of specific *VAV2* and *VAV3* gene polymorphisms with the development of functions found in mice such as cardiovascular homeostasis, hypertension, obesity, and diabetes [77,78]. More work, however, is needed to fully address this issue.

## 8. Physiological Functions of Vav Proteins, a Problem for Potential Anti-Vav Therapies?

Recent data indicate that the inhibition of the catalytic activity of Vav proteins could be of interest to treat a large variety of pathologies such as immune dysfunctions and cancer [2,79]. For example, in the case of Vav2 and Vav3, protumorigenic roles have been described in skin cancer, head and neck cancer, and p190^BCR-ABL^-driven B cell acute lymphoblastic leukemia [80,81,82]. Roles for the third family member, Vav1, in cancer and other pathologies will be described in other reviews of the Special Issue on Vav proteins of this journal. Thus, they can be considered as potentially interesting good targets if high affinity inhibitors can eventually be developed. However, *prima facie*, it can be considered that the phenotypes described in this review can preclude the application of such therapies due to the extensive side effects they can elicit in the cardiovascular system, the skeletal muscle, and the overall metabolic homeostasis in patients. It is likely that the same problem would also arise when using inhibitors targeting downstream elements of the Vav–Rac1 axis. This issue is still a matter of investigation. However, with the available evidence, we can forecast some of the most important problems and rule out other potential side effects.

In the case of Vav2, we believe that the cardiovascular defects elicited by the inhibition of Vav2 should not represent a serious hurdle for anti-Vav2 therapies for a number of reasons: (i) Genetic evidence indicates that the hypertensive state is rescued when the activity of Vav2 is restored in Vav2-deficient mice [36]; (ii) these defects can be prevented using standard anti-hypertension therapies [33,34,36]; and (iii) using homozygous *Vav2*^L332A/L332A^ (which display a reduction in 70% of Vav2 catalytic activity in tissues) and heterozygous *Vav2*^L332A/–^ (which displayed a reduction of 85% of Vav2 catalytic activity in tissues) pharmaco-mimetic mice, we have shown that the cardiovascular defects only arise in the latter animals [38]. However, effective anti-tumoral effects were obtained when using both *Vav2*^L332A/L332A^ and *Vav2*^L332A/–^ mice [38]. These results indicate that it could be possible to find therapeutic windows in which the positive anti-tumoral effects of the catalytic activation of Vav2 can be dissociated from the negative side effects [38]. Skeletal muscle defects do arise in *Vav2*^L332A/L332A^ mice though [39], thereby suggesting that the use of Vav2 inhibitors could eventually lead to the loss of muscle mass and the development of a metabolic syndrome condition if administered during very long periods of time. The time-window in which most of these defects arise, however, suggest that patients will not develop such side effects under the normal protocols usually utilized during anti-tumoral treatments [39].

In the case of Vav3, we consider that most defects found in *Vav3*^–/–^ mice do not represent a red flag for anti-Vav3 therapies because they arise as a consequence of the dysregulation of biological processes that take place during the embryonic or early postnatal times. Thus, it is unlikely that they will emerge when Vav3 is inhibited at older ages.

## 9. Concluding Remarks

Despite the progress made, many regulatory and functional issues remain to be addressed for these Vav family-dependent physiological processes in the near future. Thus, we still have a long way to go to understand the upstream receptors, regulatory molecules, and downstream pathways that are involved in most of the signaling processes described in this review. This is important from a basic science perspective, but also from pharmacological and clinical points of view, given that this information can shed light into new therapeutic avenues for high-incidence diseases. In this context, it has been generally assumed that most phenotypes found in Vav family mouse models are Rho GTPase-dependent. However, the specific GTPases that are involved remain to be clarified in the case of most of the pathways that have been discussed here. Likewise, we cannot rule out the participation of Vav-regulated adaptor pathways, at least in some of them.

The repercussions of some of the defects found in Vav family-deficient mice at the organismal level also remain poorly characterized. For example, we do not know as yet the impact of the defects found in retinal neurons on the vision of Vav family-deficient mice. As indicated above, the physiological problems associated with the myelination defects found in *Vav3*^–/–^ mice are also unknown. Many questions remain to also be addressed in the case of the regulation of sympathoexcitation by the RVLM in Vav3-deficient mice and how it is affected by the inputs received from vagal afferent fibers. All these lingering problems can be solved by continuing the analyses of genetically modified mice for *Vav* family genes and for other loci encoding proteins implicated in these processes.

Finally, we cannot rule out the implication of Vav family proteins in additional functions in the tissues and systems discussed in this review. It is also possible that additional phenotypes will be found when the genetic manipulation of *Vav* family loci is conducted in combination with other Rho GEF-encoding genes. Arguably, more work has to be done to eventually solve all these questions.

## Data Availability

Not applicable.
